# Selective disruption of *Tcf7l2* in the pancreatic β cell impairs secretory function and lowers β cell mass

**DOI:** 10.1093/hmg/ddu553

**Published:** 2014-10-29

**Authors:** Ryan K. Mitchell, Angeles Mondragon, Lingling Chen, James A. Mcginty, Paul M. French, Jorge Ferrer, Bernard Thorens, David J. Hodson, Guy A. Rutter, Gabriela Da Silva Xavier

**Affiliations:** 1 Section of Cell Biology, Division of Diabetes, Endocrinology and Metabolism, Department of Medicine,; 2 Photonics Group, Department of Physics and; 3 Section of Genetics and Medicine, Division of Diabetes, Endocrinology and Metabolism, Department of Medicine, Imperial College London, London, UK; 4 Center for Integrative Genomics, Physiology Department, University of Lausanne, Genopode Building, CH-1015 Lausanne, Switzerland

## Abstract

Type 2 diabetes (T2D) is characterized by β cell dysfunction and loss. Single nucleotide polymorphisms in the T-cell factor 7-like 2 (*TCF7L2*) gene, associated with T2D by genome-wide association studies, lead to impaired β cell function. While deletion of the homologous murine *Tcf7l2* gene throughout the developing pancreas leads to impaired glucose tolerance, deletion in the β cell in adult mice reportedly has more modest effects. To inactivate *Tcf7l2* highly selectively in β cells from the earliest expression of the *Ins1* gene (∼E11.5) we have therefore used a *Cre* recombinase introduced at the *Ins1* locus. *Tcfl2^fl/fl^::Ins1Cre* mice display impaired oral and intraperitoneal glucose tolerance by 8 and 16 weeks, respectively, and defective responses to the GLP-1 analogue liraglutide at 8 weeks. *Tcfl2^fl/fl^::Ins1Cre* islets displayed defective glucose- and GLP-1-stimulated insulin secretion and the expression of both the *Ins2* (∼20%) and *Glp1r* (∼40%) genes were significantly reduced. Glucose- and GLP-1-induced intracellular free Ca^2+^ increases, and connectivity between individual β cells, were both lowered by *Tcf7l2* deletion in islets from mice maintained on a high (60%) fat diet. Finally, analysis by optical projection tomography revealed ∼30% decrease in β cell mass in pancreata from *Tcfl2^fl/fl^::Ins1Cre* mice. These data demonstrate that *Tcf7l2* plays a cell autonomous role in the control of β cell function and mass, serving as an important regulator of gene expression and islet cell coordination. The possible relevance of these findings for the action of *TCF7L2* polymorphisms associated with Type 2 diabetes in man is discussed.

## Introduction

A considerable body of evidence suggests there is a strong hereditary component to type 2 diabetes (T2D) (1–3). Indeed, genome-wide association studies (GWAS) have now identified over 90 *loci* that are associated with T2D risk (reviewed in 4). Most of the identified single nucleotide polymorphisms (SNP) associated with T2D appear to affect β cell mass or function (5). However, most of these are in intronic or intergenic regions, making it difficult to identify the causal gene(s) and thus the impact of the identified SNP(s) at the molecular and cellular level (6).

Recently, much effort has been devoted to elucidating how the T2D-associated SNP rs7903146, which lies in intron 3 of the T-cell factor 7 like-2 (*TCF7L2*) gene, may lead to increased risk of diabetes. First described in Icelandic, Danish and US cohorts (7) SNP rs7903146 is presently the most strongly associated of the GWAS-identified variants with T2D (7–10) and is also associated with latent autoimmune diabetes in adults (11). Available clinical evidence points to an action of the risk alleles to impair insulin secretion in man with little, or a slightly protective effect, on insulin action (7–19).

TCF7L2 is a member of the TCF family of transcription factors involved in the control of cell growth and signalling downstream of wingless-type MMTV integration site family (Wnt) receptors (20) and was previously best known for its association with prostate and colon cancer development (21,22). Activation of the Wnt pathway leads to release of catenin from an inhibitory complex and its translocation to the nucleus, where it binds TCF7L2 and other related TCF factors (23). The function of this transcriptional complex is context dependent, i.e. it may act as either a transcriptional activator or repressor (5,23). In the pancreas, TCF7L2 and the Wnt signalling pathway are essential for proliferation of the pancreatic epithelium (24) and enhanced Wnt signalling has been shown to lead to islet proliferation (25). While loss of β-catenin signalling leads to pancreatic hypoplasia (26), stabilization of β-catenin results in the formation of large pancreatic tumours (27).

Studies from our own laboratory and others' have shown that silencing of *Tcf7l2* gene expression in clonal cell lines (28) and primary islets (28,29) leads to increased apoptosis and impaired β cell function. Moreover, *Lox*P-mediated deletion specifically in the pancreas using a PDX1.*Cre* (30), led to glucose intolerance and impaired β cell mass on a high fat diet. On the other hand, a recent report (31) indicated that whereas deletion in the liver led to lowered hepatic glucose output, consistent with earlier findings of perinatal mortality in global *Tcf7l2* null mice (32), deletion in the β cell in adult mice using a tamoxifen-inducible rat insulin promoter 2-driven (RIP2.Cre-ERT2) deleter strain exerted no apparent effect on glucose homeostasis. The authors therefore concluded that changes in *Tcf7l2* expression in the β cell in man are unlikely to contribute to diabetes risk. However, the latter studies were limited to the examination of relatively young (<12 weeks old) mice maintained on a normal chow diet. Moreover, deletion in adults precluded examination of the effects on β cell proliferation during early post-natal growth. Finally, it was unclear in these studies whether *Tcf7l2* expression was affected in the hypothalamus of the resulting KO mice, as might be expected using the Pdx1.*Cre* line (33).

Gene expression analysis following *Tcf7l2* deletion or silencing revealed changes in the expression of a number of genes in mouse pancreatic islets, including that encoding the GLP-1 receptor (*Glp1r*; (15,28,30,34). Correspondingly, it has previously been reported that TCF7L2 may mediate GLP-1-induced β cell proliferation (35). Since GLP-1 has also been implicated in β cell survival, the increased incidence of apoptosis in TCF7L2-silenced islets (29,36), and in individuals carrying the risk variants of TCF7L2 (15), are both consistent with lowered GLP-1 signalling (15,36). Supporting this view is the decrease in β cell mass in high fat-fed, pancreas-specific *Tcf7l2* null mice (30) and in mice over-expressing a dominant negative form of *Tcf7l2* in β cells (37). Thus, the diminished insulinotropic effect of GLP-1 in islets lacking *Tcf7l2* activity seems likely to be due, at least in part, to a lack of cognate receptors on the cell surface (28,30,37). Diminished brain GLP-1 signalling in mice over-expressing a dominant negative form of *Tcf7l2* was also reported to lead to impaired glucose tolerance and insulin sensitivity when mice were administered a high fat diet (38).

While the above evidence suggests that loss of *Tcf7l2* from the β cell is likely to impair insulin production, and hence increase T2D risk, adult *Tcf7l2* knockout mice show reduced hepatic glucose production during fasting and improved glucose homeostasis when maintained on a high fat diet (31); loss of Tcf7l2 signalling in the liver is associated with lowered expression of genes involved in glucose metabolism in this tissue (31,39). Such data together indicate that *lowering* Tcf7l2 activity, at least in the liver, may be beneficial in metabolic diseases. Moreover, it has also been reported that transgenic mice over-expressing *TCF7L2* systemically have impaired glucose homeostasis, among other physiological anomalies (40). The latter data are consistent with those from Gaulton et al. (41), indicating that chromatin at the *TCF7L2* gene is in an islet-specific ‘open’ conformation, and that in β cell lines the enhancer activity of the at-risk T-allele is elevated compared with the C-allele. Moreover, Savic et al. (42) have identified tissue-specific enhancer activity within the association interval of rs7903146 which may lead to the generation of different splice variants of *TCF7L2*, potentially leading to different functional effects in different tissues. Thus, the apparent discordance between the metabolic phenotype of the various mouse models could be partly due to the involvement of *TCF7L2* in glucose homeostasis in more than one tissue, potentially in opposing directions. Further complicating the potential actions of genomic risk loci, *TCF7L2* is subject to tissue-specific alternative splicing (43–46).

Given the existing controversy in the literature over the relative importance of *Tcf7l2* in the β cell versus the liver and other tissues (30,31), and the contributions of extrapancreatic tissues to the action of risk variants on diabetes risk (47), the present study was designed to achieve highly selective deletion of *Tcf7l2* in the β cell, from the earliest possible stages in the establishment of a definitive β cell status, i.e. expression of the insulin gene. To this end we have used a highly β cell selective *Cre* recombinase, based on expression of the enzyme from the *Ins1* locus. In contrast to the use of Pdx1- (30) or Pdx1ER-based *Cre*s (31) this strategy allows recombination highly selectively and early in the development of individual β cells, without the complications of deletion in other tissues including the brain (33,48).

We show (a) that β-cell deficient *Tcf7l2* mice show impaired insulin release and glucose homeostasis, particularly in response to oral glucose challenge; (b) when examined in intact islets, an unsuspected action is observed on both intracellular calcium signalling and cell–cell communication, likely to contribute to the deranged insulin secretion *in vivo*. These findings provide further evidence for the β-cell autonomous role of *Tcf7l2*, and imply that this factor maybe particularly important during expansion of these cells in the post-natal period or during adaptation to metabolic stresses including high fat diet

## Results

### Abnormal glucose tolerance and insulin secretion in β cell selective Tcf7l2 null mice

In the present study, we used Ins1.*Cre* mice (48) (B. Thorens and J. Ferrer, manuscript in preparation) to effect highly selective and early (∼E11.5) (49,50) deletion of *Tcf7l2* in β cells, based on *Cre* expression driven by the *Ins1* gene (33,51). The resultant *Tcf7l2* null (*Tcf7l2^fl/fl^::Ins1.Cre^+^*) mice were born at the expected Mendelian ratio and gained weight similarly to wild-type mice (*Tcf7l2^fl/fl^::Ins1.Cre^−^*) when maintained on regular chow (Supplementary Material, Fig. S1).

**Figure 1.**
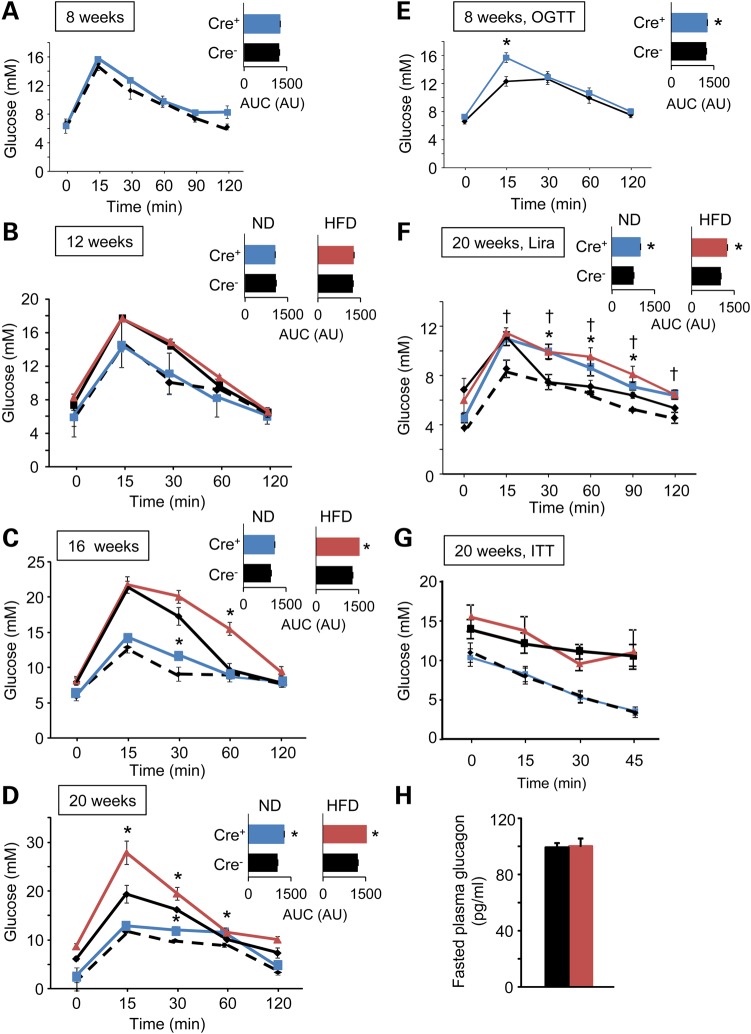
Glucose and insulin tolerance in *Tcf7l2^fl/fl^::Ins1.Cre^+^*mice. Intraperitoneal glucose tolerance was assessed at 8 (**A**), 12 (**B**), 16 (**C**) and 20 (**D**) weeks of age in *Tcf7l2^fl/fl^::Ins1.Cre^+^*(blue) and *Tcf7l2^fl/fl^::Ins1.Cre^−^* (black dotted) mice maintained on a normal chow diet (ND), and *Tcf7l2^fl/fl^::Ins1.Cre^+^*(red) and *Tcf7l2^fl/fl^::Ins1.Cre^−^* (black) maintained on a high fat (HFD; 60%) diet. Glucose tolerance and area under the curve (inset) are shown. Oral glucose tolerance was assessed at 8 weeks in mice maintained on a normal chow diet (**E**). Intraperitoneal glucose (1 g/kg) tolerance and response to liraglutide (200 µg/kg) treatment (**F**) and insulin (0.75 units/kg) tolerance (**G**) was assessed at 20 weeks in mice maintained on a normal chow diet. (**H**) Fasting plasma glucagon concentration was measured on mice fed on a HFD (see Materials and Methods) *n* = 7–10 mice; *P ≤ 0.05.

As shown in Figure 1A–D, *Tcf7l2^fl/fl^::Ins1.Cre^+^* mice developed impaired intraperitoneal glucose tolerance in an age-dependent manner, displaying significant impairments from 16 weeks (increase in AUC of 13.6 ± 2.8%, *n* = 6 mice per genotype, *P* < 0.05). Impaired oral glucose tolerance (increase in AUC of 10.6 ± 1.3%, *n* = 6, *P* < 0.05) was apparent in younger animals (from 8 weeks; Fig. 1E). Consistent with a more marked effect via altered incretin responses, the glucose excursion prompted by elevated glucose (1 g/kg) plus the GLP-1 analogue liraglutide (200 µg/kg) (52) was particularly strongly affected by *Tcf7l2* deletion (Fig. 1F). These changes were not associated with any alteration in sensitivity to intraperitoneal insulin (Fig. 1G).

**Figure 2.**
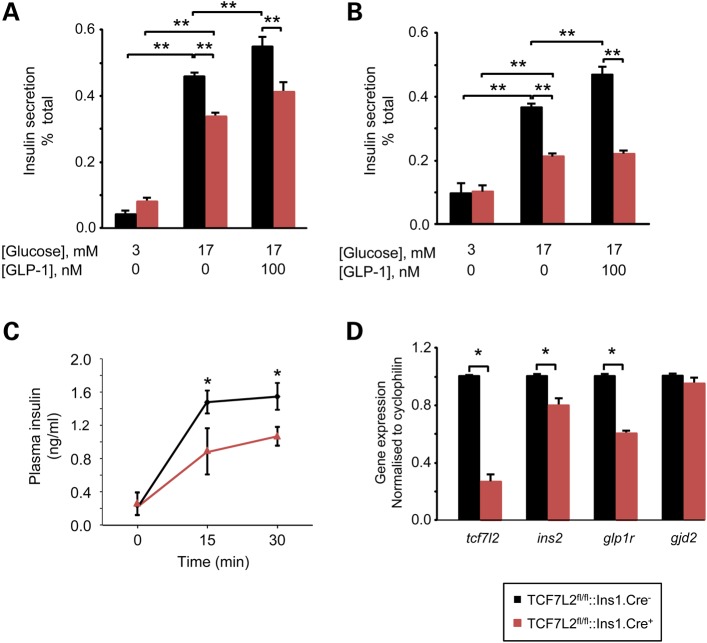
*Tcf7l2* deletion impairs glucose and GLP1-stimulated insulin secretion from isolated islets. (**A**) Plasma insulin following intraperitoneal injection of glucose from 20-week-old mice that had been maintained on a high fat diet was measured as described in Materials and Methods. (**B**) Real-time quantitative PCR analysis was performed on islets from 20-weeks-old mice that had been maintained on a normal chow diet. *n* = 7–10 mice; *P ≤ 0.05. (**C** and **D**) Insulin secretion as assessed in isolated islets from 20-week old *Tcf7l2^fl/fl^::Ins1.Cre^+^*(red) and littermate *Tcf7l2^fl/fl^::Ins1.Cre^−^* (black) mice on normal chow (C) or high fat diet (D). *n* = 5 mice per genotype; *P ≤ 0.05; **P ≤ 0.01.

To determine whether the imposition of a metabolic stress may affect the glucose intolerance observed in *Tcf7l2^fl/fl^::Ins1.Cre^+^*mice, animals were maintained for the indicated times on a high fat (∼60% total calories) diet. Null animals under these conditions gained weight similarly to wildtype littermates, albeit with a small increase versus controls apparent from 20 weeks (Supplementary Material, Fig. S1). While glucose intolerance was not apparent at 12 weeks (Fig. 1B), the dysglycemia observed at 16 and 20 weeks was further exaggerated compared with that apparent in animals maintained on regular chow (Fig. 1C and D). Insulin tolerance did not differ between genotypes (Fig. 1G), and fasting glucagon levels were identical (Fig. 1H). Nonetheless, *in vivo* insulin release prompted by IP injection of 1 g/kg glucose was markedly impaired in the null mice (Fig. 2A).

Confirming recombination in the β cell compartment, qRT-PCR revealed ∼75% decrease in the expression of *Tcf7l2* in the islet (Fig. 2B). This was associated with decreased expression of both *Ins2* and *Glp1r* (Fig. 2B).

Consistent with the impaired glucose tolerance observed *in vivo*, islets of Langerhans isolated from *Tcf7l2^fl/fl^::Ins1.Cre^+^* mice at 20 weeks displayed impaired glucose (*P* < 0.05) and GLP-1-(*P* < 0.05) stimulated insulin secretion versus islets from control *Cre*- mice (Fig. 2C). These effects were even more marked in islets extracted from high fat-diet treated animals (Fig. 2D), where the effects of GLP-1 to potentiate those of 17 mm glucose were completely abolished.

### Effects of Tcf7l2 elimination on glucose and GLP-1-induced intracellular free Ca^2+^ dynamics and β cell connectivity

**Figure 3.**
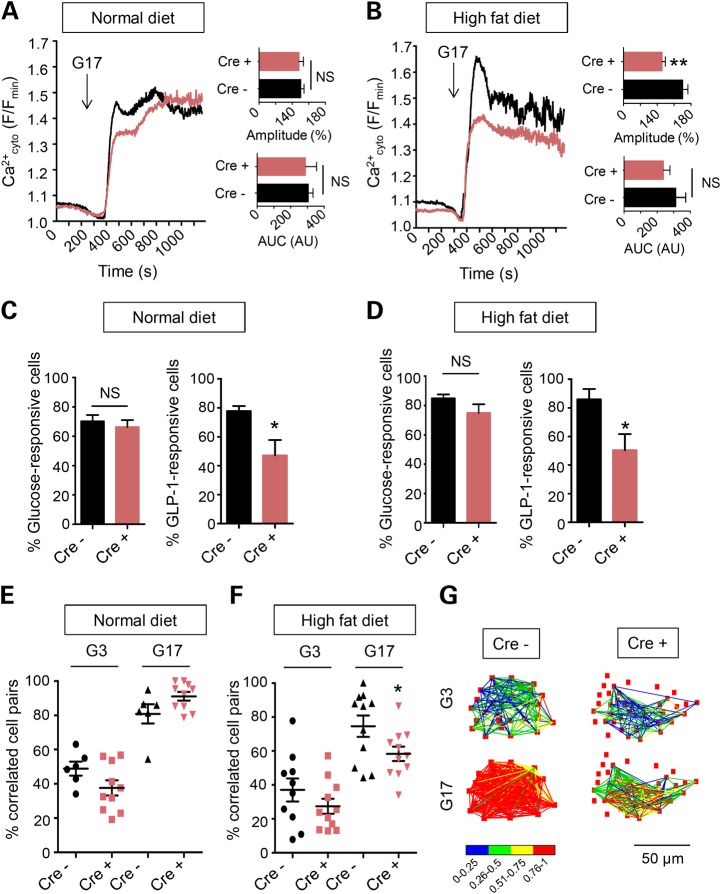
Impact of *Tcf7l2* deletion on intra- and inter-cellular Ca^2+^ dynamics. (**A** and **C**) Responses to glucose (17 mm; G17) or GLP-1 (100 nm) of β cells within intact islets of *Tcf7l2^fl/fl^::Ins1.Cre^+^*(red) and *Tcf7l2^fl/fl^::Ins1.Cre^−^* (black) mice maintained on a normal chow diet. (**B** and **D**) as for (A and C) but for islets from animals maintained for 12 weeks on a 60% fat diet. Correlation analysis (55,56) for islets from mice maintained on (**E**) normal diet and (**F**) high fat diet, and (**G**) quantitation of changes in typical islets from *Tcf7l2^fl/fl^::Ins1.Cre^+^* and *Tcf7l2^fl/fl^::Ins1.Cre^−^* mouse islets as indicated.

In healthy β cells, high glucose induces elevations in intracellular ATP/ADP ratio, the closure of ATP-sensitive K^+^ channels and the opening of voltage-gated Ca^2+^ channels to increase intracellular (cytosolic) free calcium ([Ca^2+^]_cyt_) (53,54). To further explore the mechanisms underlying the altered secretory responses in *Tcf7l2* null β cells we therefore determined whether glucose-induced [Ca^2+^]_cyt_ dynamics may be affected. Examined in whole islets from normal diet-fed mice by functional multicellular imaging (55–57), we observed largely unaltered responses to glucose of individual β cells after *Tcf7l2* elimination (Fig. 3A and C). In contrast, those in response to GLP-1 were significantly impaired, with the proportion of GLP-1-responsive cells markedly reduced (Fig. 3C). On the other hand, after maintenance on a high fat diet, both the amplitude of the [Ca^2+^]_cyt_ response to high glucose (Fig. 3B), and the proportion of cells responding to GLP-1 (Fig. 3D), were reduced in *Tcf7l2^fl/fl^::Ins1.Cre^+^* mice versus controls.

Changes in β cell ‘connectivity’, i.e. the degree to which β cells across the islet syncytium mount a coordinated (synchronized) response to stimulation (56), are associated with impaired insulin secretion (55). We have also recently demonstrated that human β cells depleted for another GWAS gene for T2D, adenylate cyclase V (*ADCY5*) (57), show impaired cellular connectivity. Whereas *Tcf7l2* elimination exerted no effect on the number of connected cell pairs in islets from normal chow-fed mice (Fig. 3E), inter-cellular connectivity was significant reduced by loss of this transcription factor from islets maintained on a high fat diet (Fig. 3F and G).

### Effects of Tcf7l2 elimination on β cell expansion in response to high fat diet

**Figure 4.**
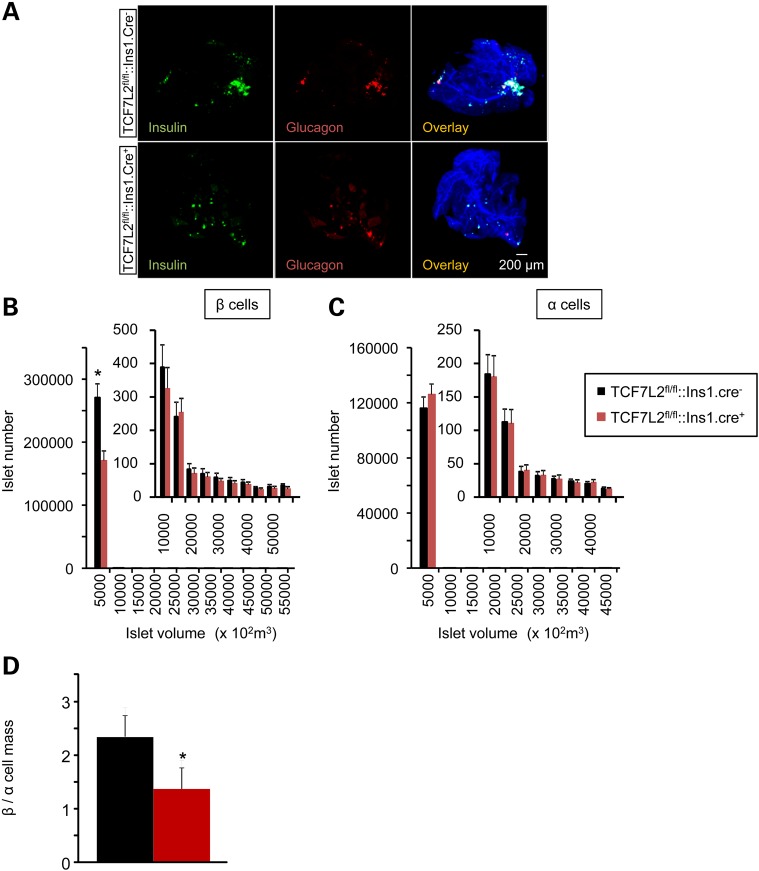
Impact of *Tcf7l2* deletion on β and α cell mass. (**A**) Representative 3D images from OPT (see Material and Methods) of *Tcf7l2^fl/fl^::Ins1.Cre^+^* and *Tcf7l2^fl/fl^::Ins1.Cre^−^* pancreata labelled with anti-insulin and anti-glucagon primary antibodies, and revealed using AlexaFluor 568 and 680, respectively. Right hand-most panels are overlaid with the autofluorescence signal revealing the pancreatic ductal system. Quantification of β (**B**), α (**C**) and (**D**) β/α cell mass from *Tcf7l2^fl/fl^::Ins1.Cre^+^*(red) and *Tcf7l2^fl/fl^::Ins1.Cre^−^* (black) knockout mice pancreata from 20-week-old mice that had been maintained on high fat diet. Quantification was conducted using Volocity software (Invitrogen), *N* = 4 mice per genotype.

To determine whether alterations in β (or α) cell mass also might contribute to impaired insulin production *in vivo* after *Tcf7l2* elimination, pancreata from 20-week-old *Tcf7l2^fl/fl^::Ins1.Cre^+^*mice that had been maintained on a HFD for 12 weeks were stained for insulin and glucagon, respectively, and analyzed by optical projection tomography (OPT). *Cre*^+^ islets displayed a markedly (31.7%, *P* < 0.05; *n* = 4 mice per genotype) decreased β cell mass, but normal α-cell mass, compared with littermate controls (Fig. 4), resulting in an overall decrease in β to α cell ratio. The number of smaller insulin-stained islets was particularly sharply decreased in *TCF7L2^fl/fl^::Ins1.Cre^+^* mouse islets (Fig. 4B).

## Discussion

The chief goal of the present studies was to reassess the role in the β cell of the Wnt signalling dependent transcription factor TCF7L2. Using a *Cre* that is highly selective for the mature β cell (48) (B. Thorens and J. Ferrer unpublished), our findings show firstly that this factor is required for (a) the normal function of these cells, and (b) their expansion under a situation of metabolic stress. Moreover, we provide novel insights into the role of *Tcf7l2* in the control of intracellular Ca^2+^ dynamics and β cell–β cell communication. The latter result was unexpected given previous findings that [Ca^2+^]_cyt_ changes were apparently unaffected by manipulation of *Tcf7l2* expression (28). However, the earlier studies were performed using isolated β cells (or small clusters of cells) rather than intact islets as examined here, where complex inter-cellular cross-talk is likely to overlay cell-intrinsic responses to stimulation. Nonetheless, the present results are consistent with very recent findings (58) showing decreased expression of the voltage-gated calcium channel subunits including *Cacna1d* in rat insulinoma-derived INS1 β cells silenced for *Tcf7l2*.

How might TCF7L2 control β cell connectivity? While the disruption of connectivity in earlier studies, e.g. after exposure to lipotoxic conditions (55), could be ascribed to impairments in the expression of the gap junction protein connexin 36 (*Gjd2*), we were unable to detect changes in *Gjd2* mRNA in *Tcf7l2* null islets in the present study (Fig. 2B), consistent with an earlier study investigating the role of Wnt signalling in the expression of other gap junction proteins (59). Changes in level or post-translational modifications (60) of this protein nonetheless remain a possibility as, of course do changes in the expression of other genes.

There is gathering evidence to suggest that the effect of TCF7L2 is mediated by resistance to incretin treatment at the level of the β cell (15,16,61–63). The present studies provide further support for this view showing both marked alterations in the expression of the *Glp1r* gene in *Tcf7l2* null islets (Fig. 2B), and impaired glucose-stimulated insulin secretion (Fig. 2C and D). Whether reduced numbers of these receptors on β cells also contributes to the apparent impairment in β cell expansion in the face of a high fat diet is unknown, but would seem worthy of future investigation.

A particularly significant finding of the present study was the appearance in *Tcf7l2^fl/fl^::Ins1.Cre^+^* mice of glucose intolerance from 16 weeks onwards demonstrating that the appearance of a β cell phenotype is dependent on age or other stresses. Importantly, in recent studies (31), the effects of *Tcf7l2* inactivation in the adult mouse β cell (using an alternative model in which exon 10, encoding the DNA-binding HMG box, was deleted) were not examined beyond 12 weeks of age, such that any effects later in life would have been missed. Of note, in the model described here, placing mice on a high fat diet increased the extent of the intolerance but did not bring forward its appearance substantially (Fig. 1).

### Relevance of the present findings for understanding of the action of TCF7L2 T2D risk variants in man

We have previously speculated (6) that variants at rs7903146 and neighbouring SNPs may have differential effects on *TCF7L2* expression the liver and on β cells as a result of differing splicing patterns in the two tissues. In particular, alternative splicing at the boundaries of exon 13, 13b and 14 leads to the loss of a ‘CRARF’ motif that appears to be selectively expressed in the β cell and susceptible to modulation by the risk T allele (34). Thus, a decrease in TCF7L2 activity at the protein level may be observed in islets from T-allele carriers despite increases in the overall levels of *TCF7L2* transcripts. Thus, the expression of multiple *Tcf7l2* alleles globally in mice (40), or forced inactivation of *Tcf7l2* in the liver (31), with its consequent dramatic effect to impair hepatic glucose output, may be of limited relevance to the action of the human SNP. In contrast, the impact of lowered *Tcf7l2* levels in the β cell, at least during the stresses imposed by aging or high fat feeding, may be more pertinent. Thus, as shown by the present studies, these are likely to act at the same time on the secretory activity of single β cells (28), the coordination of these cells within the islet (likely to further impair insulin output) (55), and finally on β cell mass. Nonetheless, further studies exploring in detail the relationship between Tcf7l2 action in different tissues would seem to be warranted.

On a final note, ‘mice are not men’ and that, given recent findings in relation to the consequences of *Slc30a8* deletion (64,65), a certain degree of circumspection is required in transposing findings from one to the other. There is currently no compelling evidence, other than immediate genomic proximity, tying *TCF7L2* to the T2D-associated variants, and it remains formally possible, though rather unlikely, that the GWAS signal is mediated via another gene. Our new study reinforces the evidence (though does not prove) that TCF7L2 is the causal gene at this locus.

## Material and Methods

### Materials

All general chemicals and tissue culture reagents were purchased from Sigma (Dorset, UK) or Invitrogen (Paisley, UK), unless otherwise stated in the text.

### Animals

All *in vivo* procedures were approved by the UK Home Office according to the Animals (Scientific Procedures) Act 1986 and were performed at the Central Biomedical Service, Imperial College, London, UK Rodents were culled by cervical dislocation. Mice were housed at two to five animals per cage in a pathogen-free facility with a 12-h light–dark cycle. Animals were fed *ad libitum* with a standard mouse chow diet (Research Diet, New Brunswick, NJ) unless otherwise stated. For high fat diet treatment, mice were placed on a high fat diet at 8 weeks of age for 8 weeks (60% [wt/wt] fat content; Research Diet, New Brunswick, NJ, USA) prior to analysis. Mice were weighed weekly from eight weeks.

### Generation and characterization of β cell-specific Tcf7l2 knockout mouse

Generation of mice carrying conditional knockout alleles of *Tcf7l2* (*Tcf7l2^fl/fl^*) was as described in (30). All mouse strains were maintained on a C57BL/6 background. *Tcf7l2^fl/fl^* mice were crossed with mice expressing *Cre* recombinase under the control of the Insulin 1 promoter (*Ins1.Cre* mice; (48), J. Ferrer, B. Thorens, unpublished) to generate *Tcf7l2* conditional knockout mice where exon 1 of the *Tcf7l2* gene was removed selectively by *Cre-*mediated excision in pancreatic β cells (*Tcf7l2^fl/fl^::ins1.Cre^+^*). Mice were born at the expected Mendelian ratios with no obvious abnormalities. Genotyping was performed by PCR using DNA from ear biopsies. Ablation of *Tcf7l2* gene expression from pancreatic islets was assessed by real-time quantitative PCR (qPCR) on islet RNA, as described below.

### Oral and intraperitoneal glucose tolerance test, insulin tolerance test

Glucose tolerance was assessed by oral and intraperitoneal administration of glucose (1 g/kg). Responsiveness to incretin treatment was assessed by co-injection of liraglutide [200 µg/kg (66); Bachem, Bubendorf, Switzerland] with glucose during an intraperitoneal glucose tolerance test. Mice were fasted for 16 h, with water available *ad libitum*. Glucose tolerance tests were conducted at 09:00 on each experimental day. Intraperitoneal insulin tolerance (075 Units per kg) test was performed as described in (67).

### Measurement of plasma hormones

Plasma glucagon from mice fasted for 16 h were measured using radioimmunoassay (Millipore, Watford, U.K.). Blood (200 µl) was removed by cardiac puncture from mice killed by cervical dislocation. Plasma was collected using high speed (2000 g, 5 min at 4°C) centrifugation in heparin-coated Microvette^®^ tubes containing EDTA (Sarstedt, Leicester, U.K.) with added DPP IV inhibitor (100 µM; Millipore, Watford, UK). For measurement of plasma insulin following intraperitoneal injection of glucose, blood (100 µl) was removed from the tail vein at various times points and plasma collected as described above. Plasma glucagon and insulin was measured by radioimmunoassay (Millipore).

### Quantitative real-time PCR analysis

Primers were designed using Primer Express 3.0 (ABI, CA, USA). Specificity for all primers was verified using BLAST (http://www.ncbi.nlm.nih.gov/blast/). RNA was extracted using Trizol (Invitrogen, UK). cDNA conversion was performed using High Capacity cDNA conversion kit (ABI, UK) after DNAse treatment (Ambion, TX, USA). Real-time PCR was performed on an ABI-Prism Fast 7500 device using powerSYBR reagent (ABI, UK). The primer sequences used were: *Tcf7l2* (forward) TTCCCCCTTGACCTCCTAGTC, *Tcf7l2* (reverse) GCACACGGTCAGTCCATGTT, *Ins2* (forward) AGCCCTAAGTGATCCGCTACAA, *Ins2* (reverse) CATGTTGAAACAATAACCTGGAAGA, *Glp1r* (forward) CCACGGTGTCCCTCTCAGA, *Glp1r* (reverse) ACTGCCGCCGGTATTCTCT, *Gjd2* (forward) CCCAGTCTCTGTTTTATCACCTATTCT, *Gjd2* (reverse) CGGCGTTCTCGCTGCTT.

### Insulin secretion assay

Secreted insulin from groups of six pancreatic islets of Langerhans was measured by radioimmunoassay (Linco, MA, USA) as previously described (68,69).

### Calcium imaging and correlation analysis

Calcium imaging and correlation analysis were performed as described in (55).

### Optical projection tomography and calculations of relative α and β cell mass

Whole pancreatic OPT, to 19 µm resolution, was performed as previously described (67,70). Dual labelling for insulin and glucagon was performed using anti-insulin antibody (DAKO) and anti-glucagon antibody (Sigma) revealed using Alexa Fluor 568 and 680 (Invitrogen), respectively.

### Statistical analysis

Values presented are the mean ± SEM for the number of observations indicated. Statistical significance and differences between means were assessed by a two-tailed Student's *t* test or one-way ANOVA with Bonferroni correction for multiple analyses. Linear correlations were calculated using regression analyses. Analysis was performed using Excel™, R, GraphPad Prism (GraphPad Software) and IgorPro.

## Supplementary Material

Supplementary Material is available at *HMG* online.

## Funding

G.D.S.X. thanks the European Foundation for the Study of Diabetes (EFSD) and Diabetes UK (13/0004672; Alec and Beryl Warren Award) for Project grants. G.A.R. thanks the MRC (UK) for Programme grant MR/J0003042/1, the BBSRC (UK) for a Project grant (BB/J015873/1), the Royal Society for a Wolfson Research Merit Award and the Wellcome Trust for a Senior Investigator Award (WT098424AIA). D.J.H. is a Diabetes UK R.D. Lawrence Fellow. The work leading to this publication has received support from the Innovative Medicines Initiative Joint Undertaking under grant agreement no. 155005 (IMIDIA), resources of which are composed of a financial contribution from the European Union′s Seventh Framework Programme (FP7/2007-2013) and EFPIA companies' in kind contribution (G.A.R.), and the Wellcome Trust (WT ISSF grant awarded to G.A.R. and P.M.F.). Funding to pay the Open Access publication charges for this article was provided by the Wellcome Trust.

## Supplementary Material

Supplementary Data

## References

[1] Pierce M., Keen H., Bradley C. (1995). Risk of diabetes in offspring of parents with non-insulin-dependent diabetes. Diabet. Med..

[2] Newman B., Selby J.V., King M.C., Slemenda C., Fabsitz R., Friedman G.D. (1987). Concordance for type 2 (non-insulin-dependent) diabetes mellitus in male twins. Diabetologia.

[3] Barroso I. (2005). Genetics of Type 2 diabetes. Diabet. Med..

[4] Basile K.J., Johnson M.E., Xia Q., Grant S.F. (2014). Genetic susceptibility to type 2 diabetes and obesity: follow-up of findings from genome-wide association studies. Int. J. Endocrinol..

[5] Perry J.R., Frayling T.M. (2008). New gene variants alter type 2 diabetes risk predominantly through reduced beta-cell function. Curr. Opin. Clin. Nutr. Metab. Care.

[6] Rutter G.A. (2014). Understanding GWAS genes for Type 2 diabetes. Diabet Med.

[7] Grant S.F., Thorleifsson G., Reynisdottir I., Benediktsson R., Manolescu A., Sainz J., Helgason A., Stefansson H., Emilsson V., Helgadottir A. (2006). Variant of transcription factor 7-like 2 (TCF7L2) gene confers risk of type 2 diabetes. Nat. Genet..

[8] Scott L.J., Mohlke K.L., Bonnycastle L.L., Willer C.J., Li Y., Duren W.L., Erdos M.R., Stringham H.M., Chines P.S., Jackson A.U. (2007). A genome-wide association study of type 2 diabetes in Finns detects multiple susceptibility variants. Science.

[9] Zeggini E., Weedon M.N., Lindgren C.M., Frayling T.M., Elliott K.S., Lango H., Timpson N.J., Perry J.R., Rayner N.W., Freathy R.M. (2007). Replication of genome-wide association signals in UK samples reveals risk loci for type 2 diabetes. Science.

[10] Sladek R., Rocheleau G., Rung J., Dina C., Shen L., Serre D., Boutin P., Vincent D., Belisle A., Hadjadj S. (2007). A genome-wide association study identifies novel risk loci for type 2 diabetes. Nature.

[11] Andersen M.K., Sterner M., Forsen T., Karajamaki A., Rolandsson O., Forsblom C., Groop P.H., Lahti K., Nilsson P.M., Groop L., Tuomi T. (2014). Type 2 diabetes susceptibility gene variants predispose to adult-onset autoimmune diabetes. Diabetologia.

[12] Saxena R., Voight B.F., Lyssenko V., Burtt N.P., de Bakker P.I., Chen H., Roix J.J., Kathiresan S., Hirschhorn J.N., Daly M.J. (2007). Genome-wide association analysis identifies loci for type 2 diabetes and triglyceride levels. Science.

[13] Saxena R., Elbers C.C., Guo Y., Peter I., Gaunt T.R., Mega J.L., Lanktree M.B., Tare A., Castillo B.A., Li Y.R. (2012). Large-scale gene-centric meta-analysis across 39 studies identifies type 2 diabetes loci. Am. J. Hum. Genet..

[14] Saxena R., Gianniny L., Burtt N.P., Lyssenko V., Giuducci C., Sjogren M., Florez J.C., Almgren P., Isomaa B., Orho-Melander M. (2006). Common single nucleotide polymorphisms in TCF7L2 are reproducibly associated with type 2 diabetes and reduce the insulin response to glucose in nondiabetic individuals. Diabetes.

[15] Villareal D.T., Robertson H., Bell G.I., Patterson B.W., Tran H., Wice B., Polonsky K.S. (2010). TCF7L2 variant rs7903146 affects the risk of type 2 diabetes by modulating incretin action. Diabetes.

[16] Pilgaard K., Jensen C.B., Schou J.H., Lyssenko V., Wegner L., Brons C., Vilsboll T., Hansen T., Madsbad S., Holst J.J. (2009). The T allele of rs7903146 TCF7L2 is associated with impaired insulinotropic action of incretin hormones, reduced 24 h profiles of plasma insulin and glucagon, and increased hepatic glucose production in young healthy men. Diabetologia.

[17] Le B.O., Kerr-Conte J., Gargani S., Delalleau N., Huyvaert M., Gmyr V., Froguel P., Neve B., Pattou F. (2012). TCF7L2 rs7903146 impairs islet function and morphology in non-diabetic individuals. Diabetologia.

[18] Deng S., Vatamaniuk M., Huang X., Doliba N., Lian M.M., Frank A., Velidedeoglu E., Desai N.M., Koeberlein B., Wolf B. (2004). Structural and functional abnormalities in the islets isolated from type 2 diabetic subjects. Diabetes.

[19] Yoon K.H., Ko S.H., Cho J.H., Lee J.M., Ahn Y.B., Song K.H., Yoo S.J., Kang M.I., Cha B.Y., Lee K.W. (2003). Selective beta-cell loss and alpha-cell expansion in patients with type 2 diabetes mellitus in Korea. J. Clin. Endocrinol. Metab.

[20] Jin T., Liu L. (2008). The Wnt signaling pathway effector TCF7L2 and type 2 diabetes mellitus. Mol. Endocrinol..

[21] Roose J., Clevers H. (1999). TCF transcription factors: molecular switches in carcinogenesis. Biochim. Biophys. Acta.

[22] Saadeddin A., Babaei-Jadidi R., Spencer-Dene B., Nateri A.S. (2009). The links between transcription, beta-catenin/JNK signaling, and carcinogenesis. Mol. Cancer Res..

[23] Reya T., Clevers H. (2005). Wnt signalling in stem cells and cancer. Nature.

[24] Papadopoulou S., Edlund H. (2005). Attenuated Wnt signaling perturbs pancreatic growth but not pancreatic function. Diabetes.

[25] Rulifson I.C., Karnik S.K., Heiser P.W., ten B.D., Chen H., Gu X., Taketo M.M., Nusse R., Hebrok M., Kim S.K. (2007). Wnt signaling regulates pancreatic beta cell proliferation. Proc. Natl. Acad. Sci. U. S. A..

[26] Wells J.M., Esni F., Boivin G.P., Aronow B.J., Stuart W., Combs C., Sklenka A., Leach S.D., Lowy A.M. (2007). Wnt/beta-catenin signaling is required for development of the exocrine pancreas. BMC Dev. Biol..

[27] Heiser P.W., Lau J., Taketo M.M., Herrera P.L., Hebrok M. (2006). Stabilization of beta-catenin impacts pancreas growth. Development.

[28] da Silva Xavier G., Loder M.K., McDonald A., Tarasov A.I., Carzaniga R., Kronenberger K., Barg S., Rutter G.A. (2009). TCF7L2 regulates late events in insulin secretion from pancreatic islet beta-cells. Diabetes.

[29] Shu L., Sauter N.S., Schulthess F.T., Matveyenko A.V., Oberholzer J., Maedler K. (2008). Transcription factor 7-like 2 regulates beta-cell survival and function in human pancreatic islets. Diabetes.

[30] da Silva Xavier G., Mondragon A., Sun G., Chen L., McGinty J.A., French P.M., Rutter G.A. (2012). Abnormal glucose tolerance and insulin secretion in pancreas-specific Tcf7l2-null mice. Diabetologia.

[31] Boj S.F., van Es J.H., Huch M., Li V.S., Jose A., Hatzis P., Mokry M., Haegebarth A., van den Born M., Chambon P. (2012). Diabetes risk gene and Wnt effector Tcf7l2/TCF4 controls hepatic response to perinatal and adult metabolic demand. Cell.

[32] Korinek V., Barker N., Moerer P., van D.E., Huls G., Peters P.J., Clevers H. (1998). Depletion of epithelial stem-cell compartments in the small intestine of mice lacking Tcf-4. Nat. Genet..

[33] Wicksteed B., Brissova M., Yan W., Opland D.M., Plank J.L., Reinert R.B., Dickson L.M., Tamarina N.A., Philipson L.H., Shostak A. (2010). Conditional gene targeting in mouse pancreatic ss-Cells: analysis of ectopic Cre transgene expression in the brain. Diabetes.

[34] Prokunina-Olsson L., Welch C., Hansson O., Adhikari N., Scott L.J., Usher N., Tong M., Sprau A., Swift A., Bonnycastle L.L. (2009). Tissue-specific alternative splicing of TCF7L2. Hum. Mol. Genet..

[35] Liu Z., Habener J.F. (2008). Glucagon-like peptide-1 activation of TCF7L2-dependent Wnt signaling enhances pancreatic beta cell proliferation. J. Biol. Chem..

[36] Shu L., Matveyenko A.V., Kerr-Conte J., Cho J.H., McIntosh C.H., Maedler K. (2009). Decreased TCF7L2 protein levels in type 2 diabetes mellitus correlate with downregulation of GIP- and GLP-1 receptors and impaired beta-cell function. Hum. Mol. Genet..

[37] Takamoto I., Kubota N., Nakaya K., Kumagai K., Hashimoto S., Kubota T., Inoue M., Kajiwara E., Katsuyama H., Obata A. (2014). TCF7L2 in mouse pancreatic beta cells plays a crucial role in glucose homeostasis by regulating beta cell mass. Diabetologia.

[38] Shao W., Wang D., Chiang Y.T., Ip W., Zhu L., Xu F., Columbus J., Belsham D.D., Irwin D.M., Zhang H. (2013). The Wnt signaling pathway effector TCF7L2 controls gut and brain proglucagon gene expression and glucose homeostasis. Diabetes.

[39] Norton L., Fourcaudot M., Abdul-Ghani M.A., Winnier D., Mehta F.F., Jenkinson C.P., Defronzo R.A. (2011). Chromatin occupancy of transcription factor 7-like 2 (TCF7L2) and its role in hepatic glucose metabolism. Diabetologia.

[40] Savic D., Ye H., Aneas I., Park S.Y., Bell G.I., Nobrega M.A. (2011). Alterations in TCF7L2 expression define its role as a key regulator of glucose metabolism. Genome Res..

[41] Gaulton K.J., Nammo T., Pasquali L., Simon J.M., Giresi P.G., Fogarty M.P., Panhuis T.M., Mieczkowski P., Secchi A., Bosco D. (2010). A map of open chromatin in human pancreatic islets. Nat. Genet..

[42] Savic D., Bell G.I., Nobrega M.A. (2012). An in vivo cis-regulatory screen at the type 2 diabetes associated TCF7L2 locus identifies multiple tissue-specific enhancers. PLoS One.

[43] Duval A., Rolland S., Tubacher E., Bui H., Thomas G., Hamelin R. (2000). The human T-cell transcription factor-4 gene: structure, extensive characterization of alternative splicings, and mutational analysis in colorectal cancer cell lines. Cancer Res..

[44] Le Bacquer O., Shu L., Marchand M., Neve B., Paroni F., Kerr C.J., Pattou F., Froguel P., Maedler K. (2011). TCF7L2 splice variants have distinct effects on beta-cell turnover and function. Hum. Mol. Genet..

[45] Mondal A.K., Das S.K., Baldini G., Chu W.S., Sharma N.K., Hackney O.G., Zhao J., Grant S.F., Elbein S.C. (2010). Genotype and tissue-specific effects on alternative splicing of the transcription factor 7-like 2 gene in humans. J. Clin. Endocrinol. Metab..

[46] Weise A., Bruser K., Elfert S., Wallmen B., Wittel Y., Wohrle S., Hecht A. (2010). Alternative splicing of Tcf7l2 transcripts generates protein variants with differential promoter-binding and transcriptional activation properties at Wnt/beta-catenin targets. Nucleic Acids Res..

[47] McCarthy M.I., Rorsman P., Gloyn A.L. (2013). TCF7L2 and diabetes: a tale of two tissues, and of two species. Cell Metab..

[48] Kone M., Pullen T.J., Sun G., Ibberson M., Martinez-Sanchez A., Sayers S., Nguyen-Tu M.S., Kantor C., Swisa A., Dor Y. (2014). LKB1 and AMPK differentially regulate pancreatic beta-cell identity. FASEB J.

[49] Yoshinari M., Daikoku S. (1982). Ontogenetic appearance of immunoreactive endocrine cells in rat pancreatic islets. Anat. Embryol. (Berl).

[50] Teitelman G., Lee J.K. (1987). Cell lineage analysis of pancreatic islet development: glucagon and insulin cells arise from catecholaminergic precursors present in the pancreatic duct. Dev. Biol..

[51] Tamarina N.A., Roe M.W., Philipson L. (2014). Characterization of mice expressing Ins1 gene promoter driven CreERT recombinase for conditional gene deletion in pancreatic beta-cells. Islets.

[52] Mondragon A., Davidsson D., Kyriakidou S., Bertling A., Gomes-Faria R., Cohen P., Rothery S., Chabosseau P., Rutter G., da Silva Xavier G. (2014). Divergent effects of liraglutide, exendin-4, and sitagliptin on beta-cell mass and indicators of pancreatitis in a mouse model of hyperglycaemia. PLoS One.

[53] Rutter G.A. (2001). Nutrient-secretion coupling in the pancreatic islet beta-cell: recent advances. Mol. Aspects Med..

[54] Rutter G.A. (2004). Visualising insulin secretion. The Minkowski Lecture 2004. Diabetologia.

[55] Hodson D.J., Mitchell R.K., Bellomo E.A., Sun G., Vinet L., Meda P., Li D., Li W.H., Bugliani M., Marchetti P. (2013). Lipotoxicity disrupts incretin-regulated human beta cell connectivity. J. Clin. Invest.

[56] Hodson D.J., Tarasov A.I., Gimeno B.S., Mitchell R.K., Johnston N.R., Haghollahi S., Cane M.C., Bugliani M., Marchetti P., Bosco D. (2014). Incretin-modulated beta cell energetics in intact islets of Langerhans. Mol. Endocrinol..

[57] Hodson D.J., Mitchell R.K., Marselli L., Pullen T.J., Gimeno B.S., Semplici F., Everett K.L., Cooper D.M., Bugliani M., Marchetti P. (2014). ADCY5 Couples Glucose to Insulin Secretion in Human Islets. Diabetes.

[58] Zhou Y., Park S.Y., Su J., Bailey K., Ottosson-Laakso E., Shcherbina L., Oskolkov N., Zhang E., Thevenin T., Fadista J. (2014). TCF7L2 is a master regulator of insulin production and processing. Hum. Mol. Genet.

[59] van der Heyden M.A., Rook M.B., Hermans M.M., Rijksen G., Boonstra J., Defize L.H., Destree O.H. (1998). Identification of connexin43 as a functional target for Wnt signalling. J. Cell Sci..

[60] Oyamada M., Takebe K., Oyamada Y. (2013). Regulation of connexin expression by transcription factors and epigenetic mechanisms. Biochim. Biophys. Acta.

[61] Schafer S.A., Tschritter O., Machicao F., Thamer C., Stefan N., Gallwitz B., Holst J.J., Dekker J.M., Hart't L.M., Nijpels G. (2007). Impaired glucagon-like peptide-1-induced insulin secretion in carriers of transcription factor 7-like 2 (TCF7L2) gene polymorphisms. Diabetologia.

[62] Lyssenko V., Lupi R., Marchetti P., Del G.S., Orho-Melander M., Almgren P., Sjogren M., Ling C., Eriksson K.F., Lethagen A.L. (2007). Mechanisms by which common variants in the TCF7L2 gene increase risk of type 2 diabetes. J. Clin. Invest.

[63] Smushkin G., Sathananthan M., Sathananthan A., Dalla M.C., Micheletto F., Zinsmeister A.R., Cobelli C., Vella A. (2012). Diabetes-associated common genetic variation and its association with GLP-1 concentrations and response to exogenous GLP-1. Diabetes.

[64] Flannick J., Thorleifsson G., Beer N.L., Jacobs S.B., Grarup N., Burtt N.P., Mahajan A., Fuchsberger C., Atzmon G., Benediktsson R. (2014). Loss-of-function mutations in SLC30A8 protect against type 2 diabetes. Nat Genet..

[65] Rutter G.A., Chimienti F. (2014). SLC30A8 mutations in type 2 diabetes. Diabetologia.

[66] Noyan-Ashraf M.H., Momen M.A., Ban K., Sadi A.M., Zhou Y.Q., Riazi A.M., Baggio L.L., Henkelman R.M., Husain M., Drucker D.J. (2009). GLP-1R agonist liraglutide activates cytoprotective pathways and improves outcomes after experimental myocardial infarction in mice. Diabetes.

[67] Sun G., Tarasov A.I., McGinty J., McDonald A., da Silva Xavier G., Gorman T., Marley A., French P.M., Parker H., Gribble F. (2010). Ablation of AMP-activated protein kinase alpha1 and alpha2 from mouse pancreatic beta cells and RIP2.Cre neurons suppresses insulin release in vivo. Diabetologia.

[68] Ainscow E.K., Zhao C., Rutter G.A. (2000). Acute overexpression of lactate dehydrogenase-A perturbs beta-cell mitochondrial metabolism and insulin secretion. Diabetes.

[69] da Silva Xavier G., Leclerc I., Varadi A., Tsuboi T., Moule S.K., Rutter G.A. (2003). Role for AMP-activated protein kinase in glucose-stimulated insulin secretion and preproinsulin gene expression. Biochem. J..

[70] Alanentalo T., Asayesh A., Morrison H., Loren C.E., Holmberg D., Sharpe J., Ahlgren U. (2007). Tomographic molecular imaging and 3D quantification within adult mouse organs. Nat. Methods.

