# Supplementary material for: Selective disruption of Tcf7l2 in the pancreatic β cell impairs secretory function and lowers β cell mass
Source: Hum Mol Genet. 2014 Oct 29;24(5):1390–9. doi: 10.1093/hmg/ddu553 (PMC4321446; doi:10.1093/hmg/ddu553)
Supplement: Supplementary Data [file supp_24_5_1390__index.html]

Selective disruption of Tcf7l2 in the pancreatic β cell impairs secretory function and lowers β cell mass — Selective disruption of Tcf7l2 in the pancreatic β cell impairs secretory function and lowers β cell mass — Supplementary Data 

# Selective disruption of *Tcf7l2* in the pancreatic β cell impairs secretory function and lowers β cell mass

## Supplementary Data

Supplementary Data

**Files in this Data Supplement:**

- Supplementary Data - Docx file
- Supplementary Figure 1 - tif file
