# Supplementary figures and images for: Selective disruption of Tcf7l2 in the pancreatic β cell impairs secretory function and lowers β cell mass
Source: Hum Mol Genet. 2014 Oct 29;24(5):1390–9. doi: 10.1093/hmg/ddu553 (PMC4321446; doi:10.1093/hmg/ddu553)

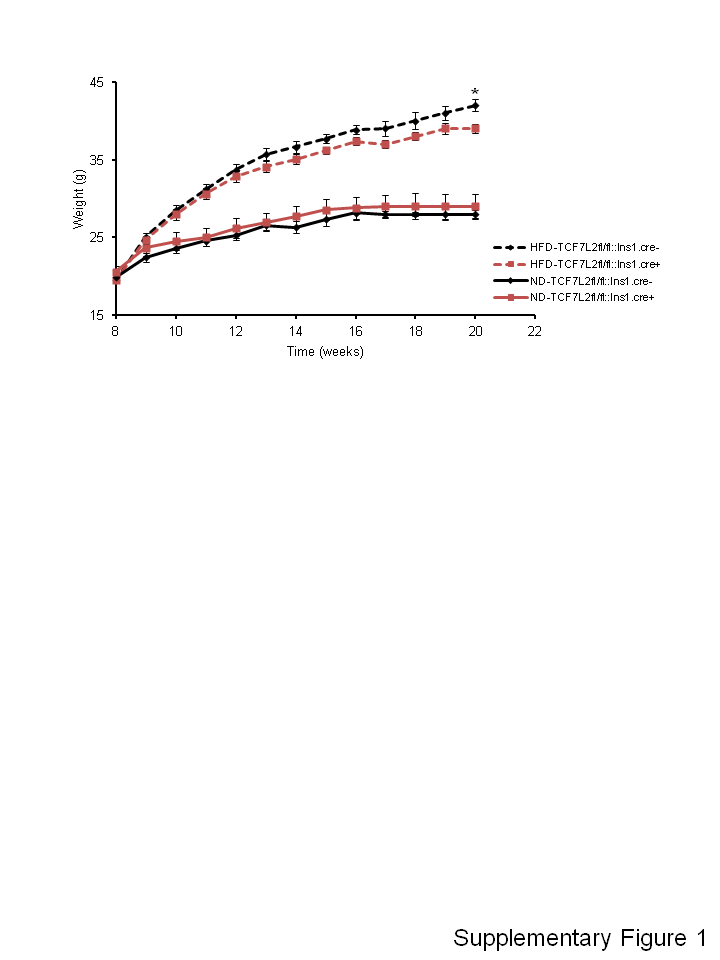

Supplement: Supplementary Data [file supp_ddu553_ddu553supp_fig1.tif]
